# Effect of non-adhering dressings on promotion of fibroblast proliferation and wound healing *in vitro*

**DOI:** 10.1038/s41598-019-40921-y

**Published:** 2019-03-13

**Authors:** Cornelia Wiegand, Martin Abel, Uta-Christina Hipler, Peter Elsner

**Affiliations:** 1 Department of Dermatology, University Hospital Jena, Jena, Germany; 2 Lohmann & Rauscher GmbH & Co. KG, Neuwied, Germany

## Abstract

Non-adhering dressings are commonly used during granulation, tissue formation, and re-epithelialization. Elucidating cytotoxic effects and influence on proliferation/migration capacity of cells like fibroblasts is of interest. Dressings’ effects were investigated by comprehensive *in vitro* approach: (1) MTT assay measuring cell viability after direct contact, (2) ATP assay determining effects on cell proliferation, and (3) scratch wound assay featuring an *in vitro* wound healing model. One cotton-based dressing with vaseline (vas) was included in the study and four polyester dressings containing vas and technology-lipido-colloid matrix (TLC), carboxymethylcellulose (CMC), hydrocolloid (HC), or glycerin (gly) as additives. A polyamide dressing with vas + CMC and three silicone-based dressings (AT, CC, M) were tested. Polyester + vas + CMC did not negatively affect cell viability or proliferation but it was found that fibroblast layers appeared more irregular with decreased F-actin network structure and tubulin density possibly leading to hampered scratch closure. Silicone AT, polyester + gly and polyamide + vas + CMC caused distinct cell damage. The latter two further reduced cell viability, proliferation and scratch healing. From the overall results, it can be concluded that cotton + vas, polyester + TLC, polyester + vas + HC and the silicone dressings CC and M have the potential to prevent damage of newly formed tissue during dressing changes and positively influence wound healing.

## Introduction

Treatment of acute and chronic wounds is challenging as it requires the selection of an appropriate dressing for both, the wound and the surrounding skin. While the focus of current material research is mainly the introduction of antimicrobial properties, the bactericidal activity of a wound dressing is not the only criterion that makes a dressing favorable for clinical use^1^. Dressings may stick to the wound surface due to dried drainage, ingrowths of newly formed tissue or a clammy dressing surface. This adhesion results in pain and tissue trauma during removal and can lead to a delay of healing^2^. Wound contact dressings are non-adhering dressings that are most commonly used during the phase of granulation, tissue formation, and re-epithelialization. The first example was Tulle gras introduced by Lumiere in the early 1900s^3^. Since then, the range of dressings described as non-adhering has expanded considerably. Several comparative clinical studies have been performed including non-adherent dressings such as Melolin^4^, paraffinated gauze^3^, Urgotul^5,6^, Atrauman^7^, Mepitel^8^ and others. These trials focused on safety and efficacy as well as reduction of wound pain and tissue trauma during dressing removal. Now, any dressing that is applied to a wound comes into intimate contact with cells involved in the healing process. As wound healing is a highly regulated, complex process, it involves different kinds of cell types, epidermal and dermal as well as leukocytes involved in inflammatory processes, coordinated by an array of cytokines and growth factors^9^. Fibroblasts are the key cells in the production of extracellular matrix that is required to fill the wound with newly synthesized tissue^10^. Upon tissue injury, they start to migrate into the damaged site and deposit collagen^1^. It is of interest to elucidate possible interactions between these cells and wound dressings as the function of fibroblasts is of great importance in the wound healing process. Cytotoxic effects of the wound dressings are especially important to note among the influence on cell proliferation and cell migration capacity^1,11,12^. Recently, Bernard *et al*. have shown that non-adhering dressings such as the product Urgotul may have beneficial effects not only due to the non-adherent and atraumatic characteristics but also owing to local interactions with the cells in the wound environment^13^. The use of such *in vitro* methods could help to elucidate and explain clinical observations to date. Tests that measure the cellular metabolic activity or the cellular ATP content are capable to detect toxic effects via reduction of cell metabolism and loss of proliferation capacity^11,12^. Cell reactions may also be accompanied by changes in cell morphology and structure, where cytotoxic effects lead to the loss of actin and tubulin networks^14^ while positive signals could result in an improved expression of these cell structure proteins. Finally, the mechanical scratch wounding of confluent monolayers serves as a model to study cell migration at the wound margins layer regeneration^15^. Here, a comprehensive experimental approach using different *in vitro* tests was applied to determine the effects of several non-adhering dressings on normal human dermal fibroblasts (NHDF) regarding promotion of fibroblast proliferation and wound healing *in vitro*. Nine commercially available non-adhering dressings were chosen that are regularly used in Germany for wound treatment (Table 1). The choice reflects the four possible base matrices cotton, polyester, polyamide and silicone. In addition, all dressings contain several other chemicals, which is why different impact on cell viability, proliferation, and morphology as well as wound healing can be expected.

**Table 1:** Summary of the non-adhering dressings included in the study presented.

Designation	Product/manufacturer	Description
cotton + vas	Lomatuell H, Lohmann & Rauscher	cotton tulle with a vaseline coating
polyester + TLC	UrgoTül, Urgo	non-occlusive gauze dressing with TLC (technology lipido-colloid) matrix
polyester + vas + CMC	Physiotulle, Coloplast	polyester tulle with vaseline and hydrocolloid carboxymethyl cellulose particles
polyester + vas + HC	Lomatuell Pro, Lohmann & Rauscher	polyester tulle with a coating compound made from a polymer matrix (an elastic, malleable fixing compound), Vaseline and hydrocolloidal carboxy-methylcellulose particles
polyester + gly	Atrauman, Hartmann	polyester tulle without vaseline containing glycerin and di-glycerin additives
polyamide + vas + CMC	Hydrotüll, Hartmann	polyamide tulle with a vaseline and carboxy-methylcellulose particles
silicone AT	ADAPTIC TOUCH, Systagenix	non-adhering dressing from cellulose acetate with a silicone coating
silicone CC	Cuticell Contact, BSN medical	silicone wound contact layer with a perforated transparent structure
silicone M	Mepitel, Mölnlycke Healthcare	primary wound contact dressing with silicone coating

Sample designation, corresponding product and the description of the manufacturer are given.

## Results

### Fibroblast viability

**Figure 1:**
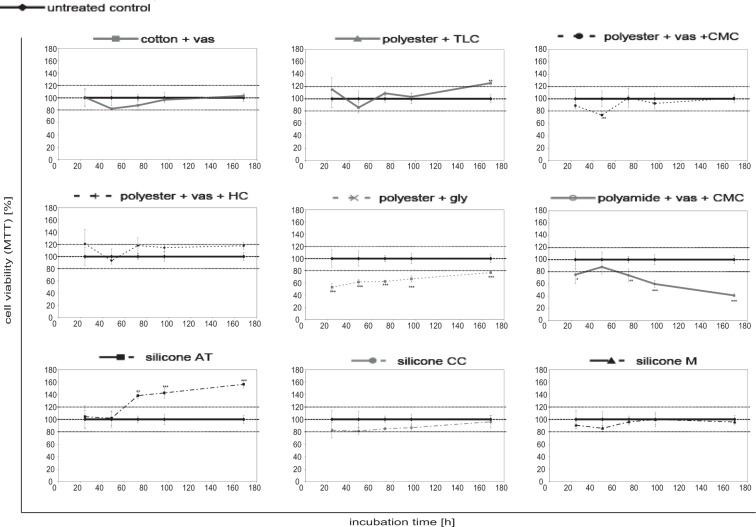
Direct effect of the different non-adhering dressings on the fibroblast viability over time. Cell viability was determined using the photometric MTT test. Data presented as mean ± standard deviation (n = 4). Asterisks indicate significant deviations from the untreated control (*p < 0.05, **p < 0.01, ***p < 0.001).

Normal human dermal fibroblasts were cultivated in 12-well-plates until they reached confluence before they were treated with the non-adhering dressing samples (1.5 cm × 1.5 cm). The dressing samples were applied directly to the fibroblast monolayer. The effect of the different non-adhering dressings on fibroblast viability was evaluated using the photometric MTT test (Fig. 1). For verification, viable fibroblasts were further quantified using the luminometric ATP assay (Supplement Fig. S1). Both tests yielded comparable results. The dressings cotton + vas and polyester + vas + HC as well as the silicone dressings CC and M exhibited no effect on cell viability, meaning that they did not alter viable cell count over the respective study period compared to the untreated control within a margin of ±20%. A slight decrease of fibroblast viability by 26.5% was observed for polyester + vas + CMC after a contact time of 48 hours (p < 0.01). However, subsequently cell viability counts returned to levels of the untreated control. In contrast, a highly significant decrease of metabolically active cells was observed for polyamide + vas + CMC and polyester + gly (p < 0.001). On the other hand, a slight increase in viable cells was observed for polyester + TLC after the end of the observational period of 168 hours (p < 0.05). This was even more pronounced for the dressing silicone AT which led to a highly significant increase in the metabolic activity in the MTT test (p < 0.001).

### Fibroblast proliferation

**Figure 2:**
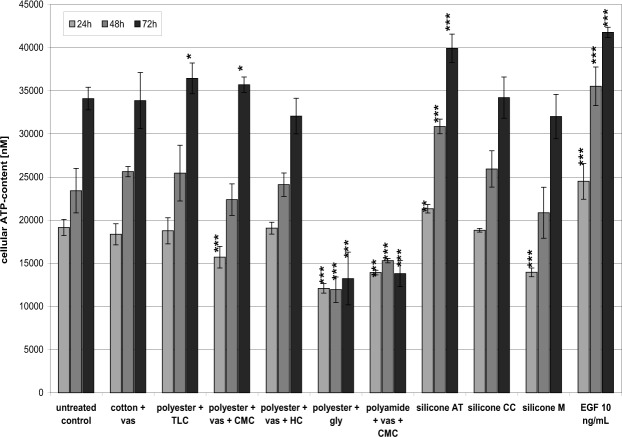
For analysis of the effect of the different non-adhering dressings on fibroblast proliferation, dressing extracts were prepared, incubated with the cells up to 72 hours, and cells quantified using the luminometric ATP assay. Effects of the dressings were compared to an untreated control and EGF-stimulated fibroblasts. Data presented as mean ± standard deviation (n = 4). Asterisks indicate significant deviations from the untreated control (*p < 0.05, **p < 0.01, ***p < 0.001).

The results of the testing of the effect on fibroblast viability suggest that some components of the non-adhering dressings might affect cell proliferation. Hence, dressing extracts were prepared, incubated with the cells up to 72 hours, and cells quantified using the luminometric ATP assay (Fig. 2) as well as the MTT test (Supplement Fig. S2). Effects of the dressings were compared to an untreated control and EGF-stimulated fibroblasts. Treatment with 10 ng/mL EGF elicited a significant increase in cell proliferation *in vitro* compared to the untreated control after 48 and 72 hours (p < 0.001). No considerable effects on the cells were observed for extracts from cotton + vas, polyester + vas + HC and silicone CC. A slight increase of cell number was observed for polyester + vas + CMC and polyester + TLC after 72 h that was statistically significant (p < 0.05). Extracts of silicone AT led to a significant increase of cell proliferation (p < 0.001). In accordance with the previous results of the MTT test, polyamide + vas + CMC and polyester + gly extracts significantly decreased cell viability and inhibited cell proliferation (p < 0.001). Moreover, the extract from dressing silicone M demonstrated a slight reductive effect after 24 hours (p < 0.001); yet, cell growth then proceeded normally. Results from ATP assay and MTT test were found to be comparable.

### Macroscopic appearance of the fibroblast monolayer and microscopic cell morphology

**Figure 3:**
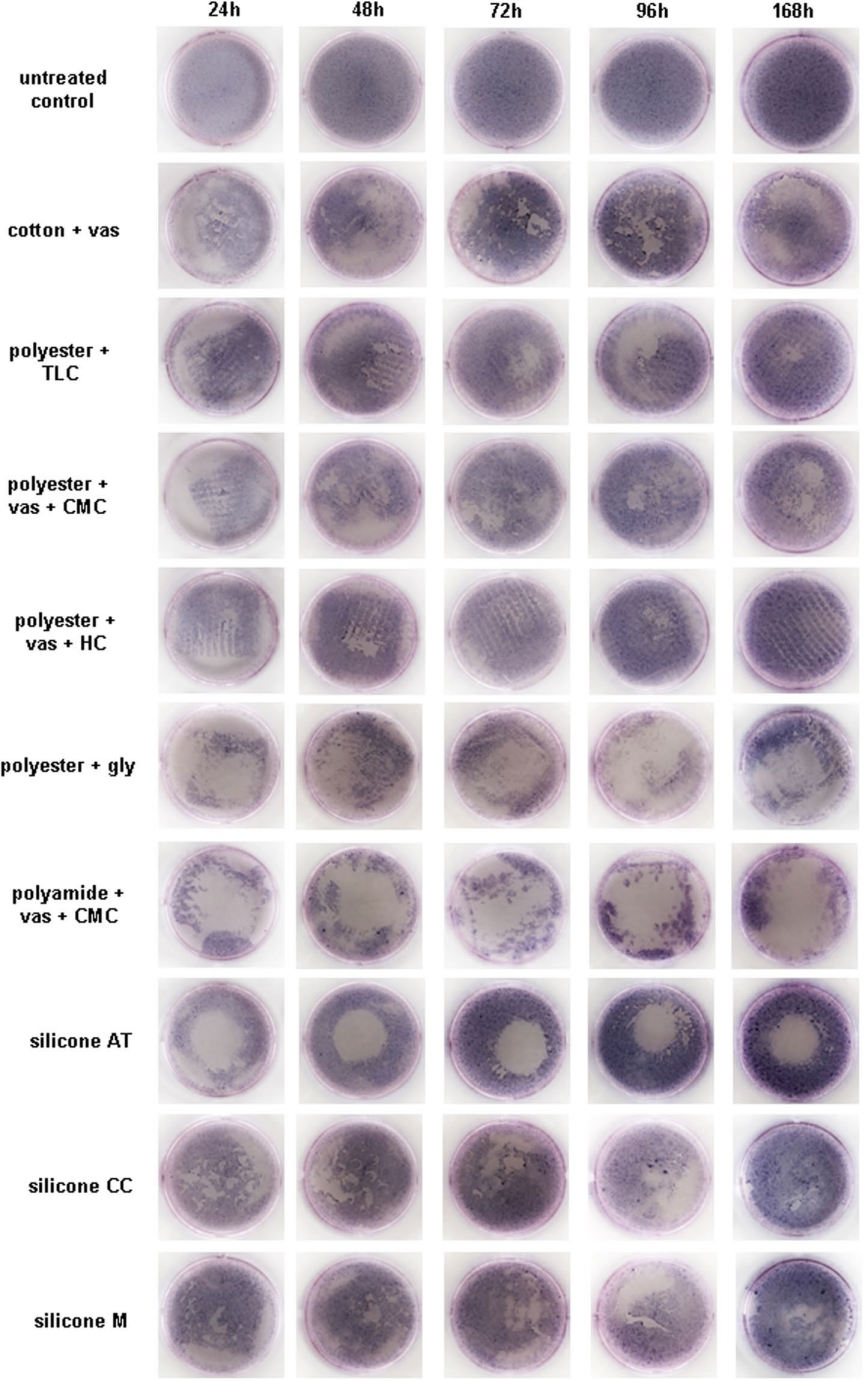
Influence of the different non-adhering dressings on the fibroblast layer was documented by taking photographs at each time point after MTT staining. Direct application of the samples led to observable injuries in the cell monolayer *in vitro* upon dressing removal while the untreated controls featured a confluent cell layer.

In general, direct application of all wound dressing samples led to observable injuries in the cell monolayer *in vitro* upon removal compared to the untreated control. The influence on the cell layer was documented by taking photographs at each time point after MTT staining (Fig. 3). Only slight changes were observed for cotton + vas and polyester + TLC or polyester + vas + CMC and polyester + vas + HC as well as the silicone dressings CC and M. In contrast, the cell monolayer underneath as well as around the samples from polyamide + vas + CMC and polyester + gly was almost completely destroyed. This is in accordance with the results for the MTT test. Surprisingly, macroscopic observations revealed that although the cell layer underneath silicone AT was almost completely abolished, a dense fibroblast layer at the well edges could be observed.

**Figure 4:**
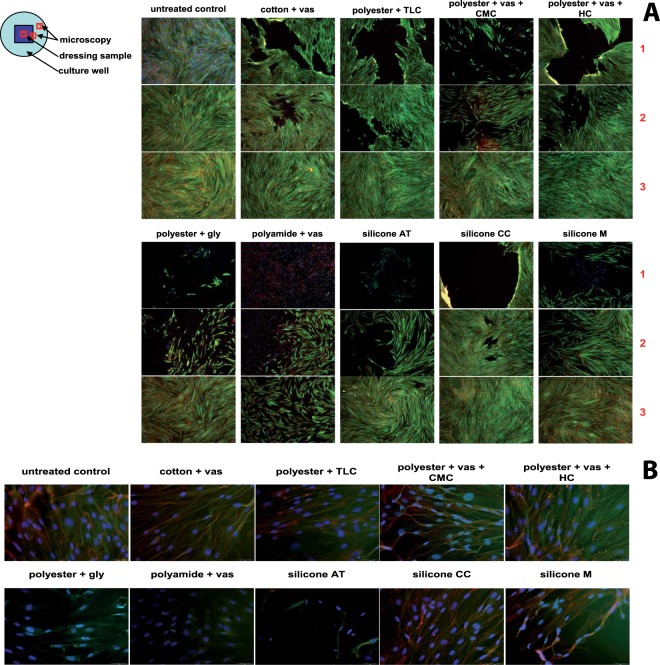
(**A**) Global impact of the non-adhering dressings on fibroblast morphology and structure after 72 hours of direct contact with the dressing samples at the positions 1 – directly underneath the dressing sample, 2 – at the margin of the dressing sample applied and the surrounding cell layer, and 3 – from the surrounding area of the cell layer. The cell nucleus is stained blue (DAPI), F-actin is dyed red (MFP TM-DY-549P1-Phalloidin), and tubulin is presented in green (anti-alpha-tubulin monoclonal antibody and Alexa Fluor^®^ 488 goat anti-mouse IgG). Magnification: 100-fold. (**B**) Surveillance of the effect underneath the non-adhering dressings (position 1) on cell morphology at 400-fold magnification.

As the macroscopic evaluation of the fibroblast monolayers after direct treatment with the dressing samples revealed somewhat unexpected results, the influence of the non-adhering dressings on cell morphology and structure was further evaluated after 72 and 168 hours by staining several cellular components like cell nucleus, actin, and tubulin. Figure 4A shows the global results after 72 hours of incubation with the dressing samples for the positions 1 – directly underneath the dressing sample, 2 – at the margin of the dressing sample applied and the surrounding cell layer, and 3 – from the surrounding area of the cell layer. Slight lesions could be found directly underneath the dressing samples in position 1 as was expected from the macroscopic evaluation (Fig. 3). Fibroblasts treated directly with samples of cotton + vas, polyester + TLC, polyester + vas + HC, and silicone CC exhibited normal cell morphology and a dense F-actin and tubulin network comparable to the untreated control (Fig. 4B). In contrast, polyester + vas + CMC and silicone M led to a loose cell arrangement and the F-actin bundles were found to be considerably less compact. After removal of the dressing silicone AT the distinct lesion in the cell layer observed macroscopically was confirmed and almost no cells were found in position 1 (Fig. 4A,B). However, at the well edges, fibroblasts in the surrounding cell layer showed distinct proliferation (Fig. 4A). Treatment with polyamide + vas + CMC and polyester + gly significantly affected cell viability and correspondingly cell morphology as a considerable destruction of the F-actin and tubulin network could be found (Fig. 4B). Images obtained after 168 hours yielded similar results (data not shown).

### Wound healing *in vitro*

**Figure 5:**
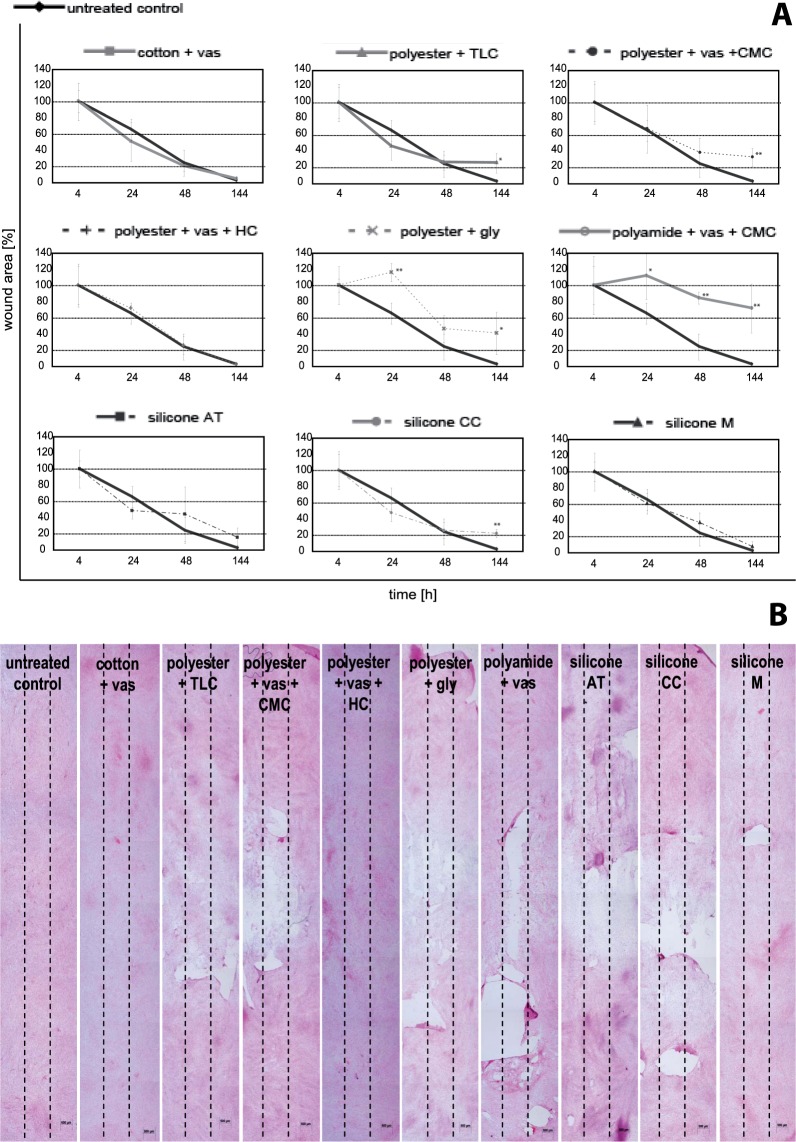
(**A**) Scratch wound healing with fibroblasts for the different non-adhering dressings presented as reduction of the wound area in [%]. Data presented as mean ± standard deviation (n = 4). Asterisks indicate significant deviations from the untreated control at the respective time point: *p < 0.05 and **p < 0.01. (**B**) Almost complete scratch closure was found with cotton + vas and polyester + vas + HC after 144 hours. Neither did polyester + vas + CMC and polyester + TLC as well as the silicone dressings CC and M affect the fibroblast scratch healing *in vitro* negatively. With polyamide + vas + CMC and polyester + gly scratches remained open during test period. Cells were stained with haematoxylin-eosin. Dotted lines represent the initial scratch width.

The influence of the non-adhering dressings on wound healing was evaluated using an *in vitro* scratch wound assay with fibroblasts. The mechanical scratch wounding of confluent monolayers serves as a model to study cell migration at the wound margins and allows the direct measurement of cell migration and layer regeneration by cell proliferation^16,17^. Encouraged by the demonstration of direct application of samples of non-adhering dressings to fibroblasts monolayers by Bernard *et al*. and the results of this study, it was decided to use the scratch wound assay with direct application of the dressing samples. *In vitro* wound closure was investigated at 4, 24, 48, and 144 hours post injury time. The untreated control exhibited complete scratch healing over the time period of 144 hours which can be quantified by the residual scratch width. Data obtained for the non-adhering dressings tested on scratch wound healing was transformed from scratch width in [pixel] to wound area in [%] (Fig. 5A). For comparison, slopes for healing progression were further obtained by linear regression (Table 2). Measurement of the initial scratch width after 4 hours displayed no significant differences between the starting values for each dressing. It was found that cotton + vas, polyester + vas + HC, and silicone M exhibited a positive healing progression and almost complete scratch closure after 144 hours (Fig. 5, Table 2). As expected from the previous results, polyester + vas + CMC and polyester + TLC as well as the silicone dressing CC did not affect fibroblast scratch healing *in vitro* negatively. The dressing polyamide + vas + CMC on the other hand led to a distinct inhibition of healing progression (Fig. 5A, Table 2) and scratches remained open after the test period of 144 hours (Fig. 5B). This is in accordance with previously obtained results which showed that, polyamide + vas exert a negative effect on fibroblast viability and proliferation *in vitro* (Figs 1 and 2). Although the dressing polyester + gly exhibited comparable negative results on viability and proliferation of fibroblasts in these tests (Figs 1 and 2), the healing progression was comparable to that of silicone CC and polyester + TLC or polyester + vas + CMC (Table 2). However, a distinct delay in scratch closure until 24 hours was found for polyester + gly (Fig. 5A) and the scratches were still open over the examined time course (Fig. 5B). The expected adverse impact on the cell layer underneath the dressing silicone AT was not observed (Fig. 5, Table 2). Surprisingly, silicone AT demonstrated no negative influence on the healing of the scratches, although it damaged confluent cell monolayers distinctly (Fig. 3).

**Table 2:** Comparison of the scratch healing progression over time underneath the different non-adhering wound dressings by calculation of the slope through linear regression.

sample	slope for healing progression
untreated control	−0.6136
cotton + vas	−0.5558
polyester + TLC	−0.3843
polyester + vas + CMC	−0.3958
polyester + vas + HC	−0.6355
polyester + gly	−0.4671
polyamide + vas + CMC	−0.2404
silicone AT	−0.4805
silicone CC	−0.4805
silicone M	−0.5703

## Discussion

Traditional dressings may adhere to wounds resulting in significant pain and trauma to newly formed tissue upon removal. The development of non-adhering dressings has addressed this problem. To render basic materials such as cotton, polyester and polyamide less adherent, finishes such as vaseline and glycerine as well as carboxymethyl or polyacrylamide hydrogel layers^1,7,18^. Another approach is the use of soft silicone^19^ or even waxes^20^ to prevent dressings from sticking to the wound surface. Successful relying of decreased adherence is measured by *in vitro* models based for instance on gelatine bodies and examination of removal forces after dressing appliance^21^. Yet, dressing applicability is not only described by easing detachment but further requires good cell compatibility. Cytotoxic effects of dressings applied to a wound would decrease healing rates by reducing cell viability, cell proliferation and cell migration. Dressings can be investigated for these effects by the comprehensive *in vitro* approach proposed in this study using the MTT assay for measurement of cell viability after direct contact with the samples over a prolonged time period, by applying the ATP assay for determination of effects on cell proliferation over time, and employing the scratch wound assay for evaluation of the influence on wound healing. Cytoskeletal components such as actin and tubulin further play a key role in cell behavior modification especially under stress^13,22^. When a dressing is applied to the wound, it comes into intimate contact with the cells involved in the healing process. This was mimicked here by direct application of the dressing samples on the cell monolayers, allowing the physical interaction of the dressing with the underlying cells. Bernard *et al*. used a similar approach but only on confluent fibroblast layers^13^ and not in combination with a scratch wound assay. Nine commercially available non-adhering dressings were investigated that are regularly used in Germany for wound treatment (Table 1). The choice reflects the four possible base matrices cotton, polyester, polyamide and silicone. In addition, all dressings contain several other chemicals, which is why different impact on cell viability, proliferation, and morphology as well as wound healing was expected.

Fibroblasts, together with keratinocytes, enable the maintenance of the barrier function of normal skin. Upon wounding, this shield is disrupted and cells migrate into the wound area for regeneration of the skin which entails remodeling of the extracellular matrix by its proteolytic degradation as well as by de novo synthesis and deposition of newly formed matrix components. Prerequisite for the adequate cellular function is a high cell viability and sufficient cell proliferation. Cytotoxic effects of dressings applied to a wound would hence hamper the healing process. The dressings cotton + vas, polyester + vas + HC and silicone M supported fibroblast scratch healing and cells demonstrated normal cell proliferation and migration, leading to an almost complete scratch closure over the time period. In accordance, cells underneath these dressings demonstrated normal cell morphology featuring a dense cell packing comparable to the control and compact F-actin bundles. Only in the case of silicone M a slightly more irregular fibroblast layer appeared was found and the network structure of F-actin and tubulin was less dense. As no lower tendency towards healing in the scratch wound assay was observed, the slightly looser cell arrangement with less compact F-actin bundles found with silicone M might likewise be a sign for actively progressing cells. Visual examination of the MTT-stained cell layers supported the high compatibility of these dressings, although slight abrasions in the fibroblast layers were noted after removal of all dressings. Similarly, no harmful effects of the silicone samples AT or CC and polyester + TLC were observed in the *in vitro* wound healing assay. However, initial application of silicone AT on confluent fibroblast layers demonstrated distinct damage of the cell layer despite the induction of a distinct cell proliferation in its proximity. It is suggested that the dressing releases a chemical with probable proliferative effects. The nature of the substance is unclear, especially as silicones have been used as negative controls in cytotoxicity studies showing no effects^23,24^. However, own observations of plastics’ effects on keratinocytes and fibroblasts have shown that plasticizer contained in the materials may induce cell proliferation (unpublished results). Bernard *et al*. observed a similar outcome using the dressing Tulle gras^13^. As the dressing extract furthermore exhibited a distinct increase in cellular proliferative activity which may have led to the rapid and almost complete closure of the scratch wound, it may be assumed that while the dressing has the potential to induce tissue regeneration, it might also hold the threat of cell damage by deduction. The distinct proliferative influence of polyester + TLC previously reported^13^ could not be confirmed here, although a slight buoyance of cell proliferation could be observed after 72 hours. The dressing polyester + vas + CMC exhibited a slight effect on fibroblast scratch closure with more than 30% of the scratch remaining open after 144 hours. Although polyester + vas + CMC did not negatively affect cell viability or cell proliferation, it was found that the fibroblast layer appeared more irregular and the network structure of F-actin and tubulin density was less dense possibly leading to the observed hampered scratch closure *in vitro*. Abnormal cell morphology might indicate toxic effects on cells and was for instance observed in keratinocytes after treatment with paraffin gauze^25^. Polyester + gly and polyamide + vas + CMC noticeably reduced cell viability and proliferation in this study. In accordance, visual inspection of the fibroblast cell layers demonstrated distinct damage with these two dressings. Microscopic evaluation could further show that normal cell morphology directly under the dressing and at the dressing margin is almost completely lost. Correspondingly, treatment with polyester + gly and polyamide + vas + CMC noticeably decreased the healing progression and the remaining wound area was determined to be 72% and 41%, respectively. This might be due to a pasting effect of the released chemicals agglutinating the cells and impeding metabolic activities.

Wound healing is a complex biological process and, hence, not easily studied *in vivo*. However, there are a few *in vitro* assays and animal models available to readily examine the influence of substances and materials on the regeneration of tissue and the progression of wound healing. Here, we suggest a comprehensive *in vitro* approach assessing cell viability, proliferation, and morphology as well as wound healing *in vitro*. Of course, these models used are limited to the use of human cell cultures and cannot take into account the complex, multicellular, and 3-dimensional interactions occurring during wound healing. Moreover, these materials would initially interact with the host’s inflammatory cells *in vivo* that would in turn provide important signaling to the dermal fibroblasts. Nonetheless, the suggested comprehensive *in vitro* approach was able to clearly discern differences in the dressings that might eventually exert a clinical effect. Overall, it could be shown that cotton + vas and polyester + TLC as well as polyester + vas + HC and the silicone dressings CC and M have the potential to prevent damage of newly formed tissue during dressing changes and positively influence the wound healing outcome. So far, corresponding comparative clinical trials are lacking. Hence, using such an *in vitro* assay array allows the comparison of the performance of already available products under standardized conditions as well as enables the technical monitoring of material developments.

## Materials and Methods

### Materials

Different non-adhering dressings were included in the study. Table 1 shows sample designation, the corresponding product and the description given by the manufacturer.

### Cultivation of dermal fibroblasts

Normal human dermal fibroblasts (NHDF, Promocell) were cultured in Dulbecco´s modified Eagle’s Medium (DMEM, AMIMED) supplemented with 1% antibiotic-antimycotic solution (AMIMED) and 10% fetal calf serum (FCS, Promocell). The cells were cultured for 7 days in 75-cm^2^ cell culture flasks (Greiner bio-one, Germany) at 37 °C and in humidified atmosphere containing 5% CO_2_ atmosphere.

### Sample preparation

Wound dressing samples were cut aseptically corresponding to 1.5 cm × 1.5 cm and specimens were a) used directly for testing (fibroblast viability, fibroblast morphology, and scratch assay) and b) were extracted prior to testing (fibroblast proliferation). Extracts of wound dressing samples were prepared by addition of 400 µL DMEM per specimen (containing 1% antibiotic-antimycotic solution) and incubation for 48 h at 37 °C. Prior to testing, extracts were further supplemented with 10% FCS.

### Determination of the influence on fibroblast viability

For experiments, fibroblasts were harvested through trypsin-EDTA (Gibco) treatment, seeded into 12-well plates (Greiner bio-one, Germany) at a density of 10,000 cells/cm^2^, and cultured for 96 h to confluence. Afterwards, fibroblasts were incubated directly with the test specimen in complete DMEM (contains 1% antibiotic-antimycotic solution and 10% FCS) for 24 to 168 h. Cells cultured in complete DMEM alone served as untreated control. Cells were evaluated microscopically for damage after the respective incubation period and photographs of the 12-well plates were taken after MTT staining for documentation of the cell layer. The number of viable, metabolically active cells was determined using the photometric MTT assay (MTT Cell Proliferation Assay Kit, Invitrogen) according to the manufacturer’s recommendations. The assay determines the conversion of the MTT substrate to a violet product. In brief, treated or control cells were incubated with substrate solution for 2 h at 37 °C. Subsequently, the supernatant was removed and isopropyl alcohol was added to each well. After incubation for 2 h, 200 µL of the cell lysates were transferred in duplicate to 96-well plates. The absorbance was measured at 580 nm using a microplate photometer (POLARstar Galaxy, BMG Labtech, Germany). The number of viable cells was further determined using a luminometric ATP assay (ATPLiteTM-M Assay, PerkinElmer). The assay measures the generation of light in the ATP-dependent conversion of luciferin by luciferase. The emitted light is directly proportional to the ATP concentration. In brief, lysis solution was added to the wells containing treated or control cells. Subsequently, substrate solution (luciferase/D-luciferin) was added to each well. After incubation, the luminescence was quantified using a microplate luminometer (LUMIstar Galaxy, BMG Labtech, Germany). ATP concentrations were calculated on the basis of a standard curve. The amount of viable cells was calculated as percent of the control.

### Investigation of the influence on fibroblast proliferation

For experiments, fibroblasts were harvested through trypsin-EDTA (Gibco) treatment, seeded into 12-well plates (Greiner bio-one, Germany) at a density of 10,000 cells/cm^2^, and cultured for 24 h to 30% confluence. Afterwards, fibroblasts were incubated with the test specimen extracts (refer to sample preparation for details) for 24 to 72 h. Cells cultured in complete DMEM served as untreated control. Additionally, cells incubated with 10 ng/mL epidermal growth factor (EGF, Promocell) were used as positive control for proliferative effects. Determination of cell proliferation was carried out using the luminometric ATP assay (ATPLiteTM-M Assay, PerkinElmer). Here, the amount of viable cells was specified by cellular ATP-content in [nM]. The number of viable, metabolically active cells was further determined using the photometric MTT assay (MTT Cell Proliferation Assay Kit, Invitrogen). The cell amounts are given as the absorbance measured at 580 nm in [mOD].

### Visualization of the effect on fibroblast morphology and structure

For experiments, fibroblasts were harvested through trypsin-EDTA (Gibco) treatment, seeded into 12-well plates (Greiner bio-one, Germany) at a density of 10,000 cells/cm^2^, and cultured for 72 h to medium confluence. Afterwards, fibroblasts were incubated directly with the test specimen in complete DMEM (contains 1% antibiotic-antimycotic solution and 10% FCS) for 72 and 168 h. Cells cultured in complete DMEM alone served as untreated control. After incubation, cells were washed, fixed and permeabilized for staining of cellular components. For evaluation of cell morphology and structure, the cell nucleus was stained using DAPI (Carl Roth; blue fluorescence), F-actin was dyed with MFP TM-DY-549P1-Phalloidin (mobitec; red fluorescence), and tubulin was detected using an anti-alpha-tubulin monoclonal antibody (T9026, Sigma) and Alexa Fluor^®^ 488 goat anti-mouse IgG (H + L) (A-11001, mobitec; green fluorescence). Microscopic evaluation was carried out using a fluorescence microscope (Axio Scope A.1, Carl Zeiss GmbH) and images were obtained with the digital camera ColorView II (Soft Imaging Systems) at the positions 1 – directly underneath the dressing sample, 2 – at the margin of the dressing sample applied and the surrounding cell layer, and 3 – from the surrounding area of the cell layer. Cells in the image section were counted using the DAPI-stained nuclei as cell reference and employing the cell counting tool of Image J 1.45 m (NIH, Bethesda, Maryland, U.S.).

### Scratch wound assay

For experiments, fibroblasts were harvested through trypsin-EDTA (Gibco) treatment, seeded into 4-well culture slides (BD Biosciences, Germany) at a density of 40,000 cells/cm^2^, and cultured for 48 h to confluence. Afterwards, fibroblasts monolayers were scratched with a sterile pipette tip and washed with medium to remove any loose cells. Then, wound dressing samples were placed directly on the scratch and weighed down with a small polypropylene (PP) weight (sterilized Nunc™ Cap Mats, ThermoScientific, Germany). Test specimens were incubated with the cells for 4, 24, 48, and 144 hours in complete DMEM (contains 1% antibiotic-antimycotic solution and 10% FCS). Cells cultured in complete DMEM alone served as untreated controls. After the respective incubation periods, dressing samples were removed and cells were stained with hematoxylin and eosin for evaluation. Microscopic evaluation was carried out using the Axio Scope A.1 microscope (Carl Zeiss GmbH, Germany) and images were obtained with the digital camera AxioCam MRc (Carl Zeiss GmbH, Germany). Scratch width was determined using the ScratchCalculator (Mario Kanka, Jena). The progression of ‘healing’ of the scratch wound, designated as wound area in [%], was calculated in percent comparing scratch width at each time point to the initial scratch width at 4 hours. Furthermore, the slope was calculated by linear regression (Microsoft ® Excel 2000) for comparison of the healing progression over time.

### Statistical analysis

Experiments were performed four times for every wound dressing. Measurements were carried out in duplicate. All values are expressed as means ± SD (standard deviation). One-way analysis of variance was carried out to determine statistical significances (Microsoft Excel 2000). Differences were considered statistically significant at a level of p < 0.05. Asterisks indicate significant deviations from the control (*p < 0.05; **p < 0.01; ***p < 0.001).

## Supplementary information

Supplementary figures
