# Supplementary material for: Effect of non-adhering dressings on promotion of fibroblast proliferation and wound healing in vitro
Source: Sci Rep. 2019 Mar 13;9:4320. doi: 10.1038/s41598-019-40921-y (PMC6416289; doi:10.1038/s41598-019-40921-y)

Supplementary Material

Effect of non-adhering dressings on promotion of fibroblast proliferation and wound healing *in vitro*

Cornelia Wiegand[1,*], Martin Abel[2], Uta-Christina Hipler[1], Peter Elsner[1]

[1]Department of Dermatology, University Hospital Jena, Germany

[2]Lohmann & Rauscher GmbH & Co. KG, Neuwied, Germany

*Corresponding author: Cornelia Wiegand, Department of Dermatology, University Hospital Jena, Erfurter Straße 35, 07740 Jena, Germany, Email: C.Wiegand@med.uni-jena.de, Tel. +49 3641 937584, Fax +49 3641 937437

Supplement figure S1: Determination of direct effect of the different non-adhering dressings on fibroblast viability over time was verified by measurement of viable cells using the luminometric ATP assay. Data presented as mean ± standard deviation (n = 4). Asterisks indicate significant deviations from the untreated control (* p < 0.05, ** p < 0.01, *** p < 0.001).

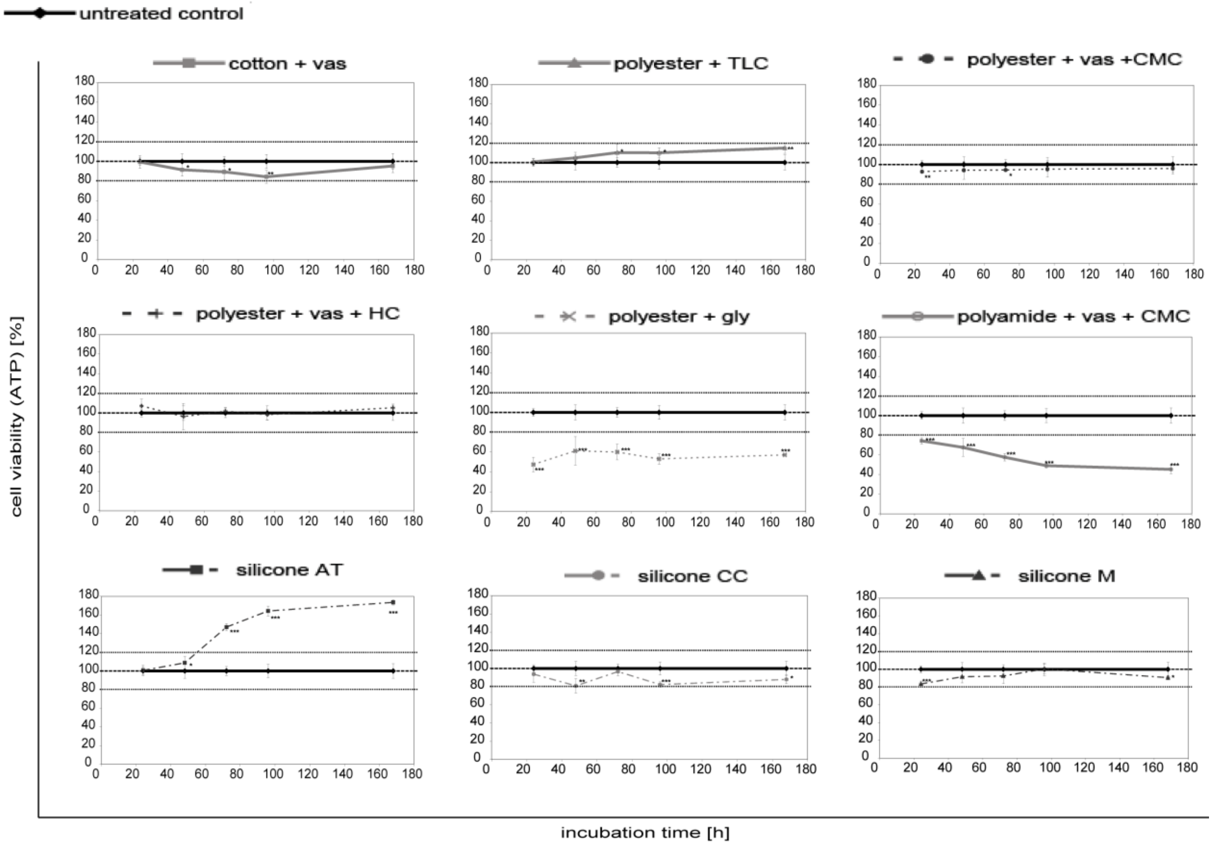

Supplement figure S2: Cell quantification was carried out using the MTT test for verification of analysis of the effect of the extracts of the non-adhering dressings on fibroblast proliferation over 72 hours. Data presented as mean ± standard deviation (n = 4). Asterisks indicate significant deviations from the untreated control (* $p < 0.05$, ** $p < 0.01$, *** $p < 0.001$).

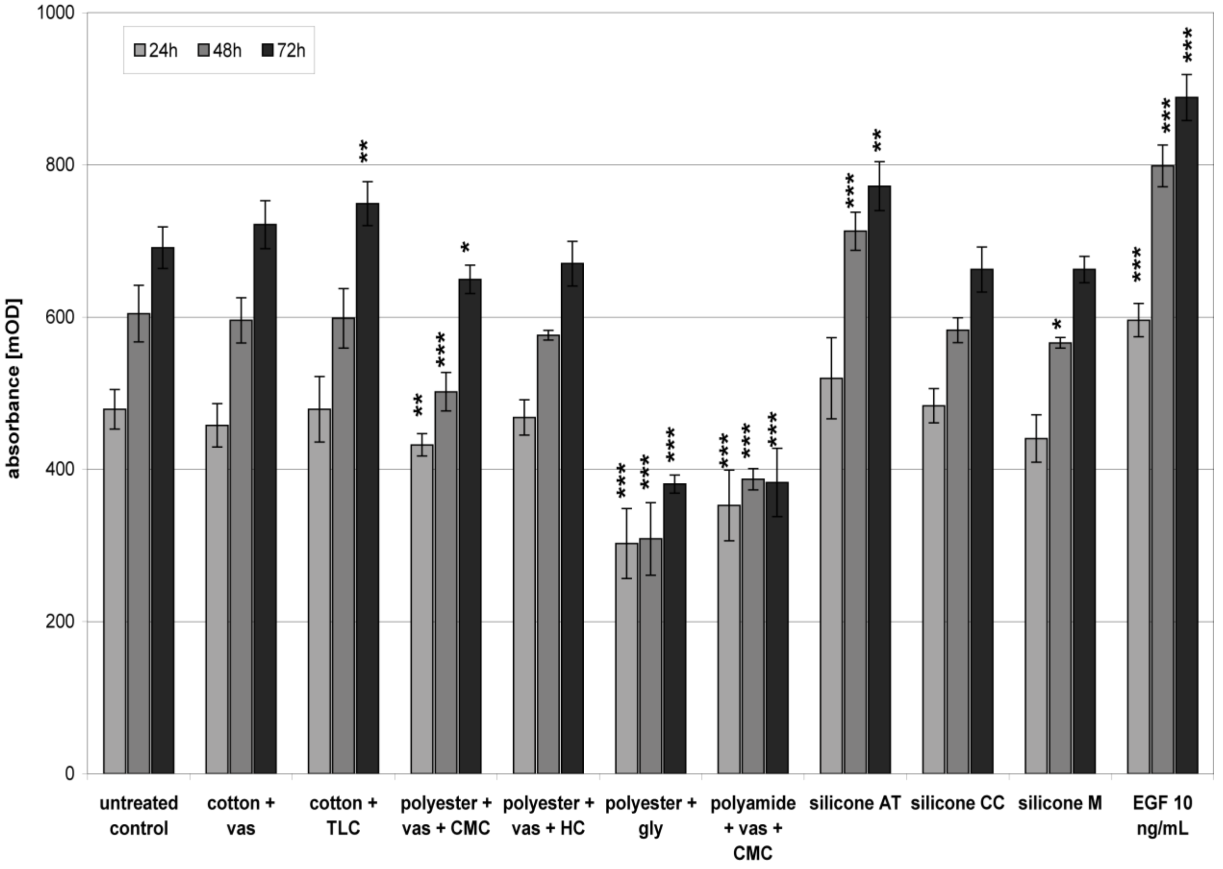

Supplement: Supplementary file 1 — Supplementary figures [file 41598_2019_40921_MOESM1_ESM.pdf]
